# Mastering the question: tips and examples for effective ophthalmology exams

**Published:** 2026-02-09

**Authors:** Anthony Green, Rebecca Ford, Luis Amaya, Richard Bowman

**Affiliations:** 1 Educational Advisor on Examinations: Ophthalmology Foundation, USA.; 2 Consultant Ophthalmologist: Bristol Eye Hospital. United Kingdom.; 3 Consultant Paediatric Ophthalmology: St Thomas’ Hospital, London, United Kingdom.; 4 Consultant Ophthalmologist: Great Ormond Street Hospital, Hon Assoc Prof: UCL Institute of Child Health and London School of Hygiene & Tropical Medicine.

**Well-constructed questions allow examiners to identify which candidates have the necessary competence to practice as ophthalmologists.**

Effective ophthalmology exam questions must be clinically relevant, fair, and well-constructed, allowing examiners to confidently distinguish between candidates who have the necessary competence and those who do not.

We recommend following a step-wise approach.

**Step 1. Overall exam design**

Start by creating a blueprint that aligns the overall exam content with the course objectives and curriculum, at the level of complexity required for the learners (e.g., medical student vs. ophthalmology fellow).

**Step 2. Review team**

Assemble a review team of subject matter experts or colleagues who understand question development and best practice.

**Step 3. Question design**

Design questions that require candidates to apply their knowledge and clinical reasoning skills, rather than simply recalling isolated facts.

- Use patient scenarios that mirror real-world situations and include only information that is relevant to the problem.
- Question types like single best answer or extended matching questions work well. They have one clearly correct option and plausible wrong answers.
- Avoid negative phrasing (e.g., “Which one is NOT a beta-blocker?”). If this is unavoidable, highlight or capitalise the negative word, as in the example above. This is to reduce the risk that the student will make a mistake that reflects their ability to read under pressure, rather than their actual level of knowledge.
- All answer options must be grammatically consistent and similar in length, so that candidates can’t easily guess the correct answer.
- Do not use absolute terms like ‘always’ or ‘never’ in the answer options – these usually suggest an incorrect answer.

Avoid using “All of the above” and/or “None of the above” as answer options like these test students’ exam strategies more than their actual understanding.

**Step 4. Review and pilot test**

After writing, the questions should undergo a formal review process by experts and potentially be pilot tested to ensure quality, difficulty, and their ability to effectively discriminate between students with higher and lower levels of understanding.

**Sample questions**

*The following questions have been peer reviewed and are of the standard used in the Ophthalmology Foundation exams.*

**1. Visual science**
  Which **ONE** of the following is most likely to be true of conditions with mitochondrial inheritance?
  - Affected fathers transmit the condition
  - They follow a recessive inheritance pattern
  - They are passed exclusively by mothers
  - They only affect males
  Answer: **c.** They are passed exclusively by mothers (because mitochondrial DNA is inherited maternally).
**2. Optics, refraction, and instruments**
  Which **ONE** of the following clinical scenarios is MOST likely to result in pseudo-neutralisation during retinoscopy?
  - Performing retinoscopy on a child without cycloplegia.
  - Performing retinoscopy under mydriasis with full cycloplegia.
  - Retinoscopy in a pseudophakic eye with a centered IOL.
  - Use of trial lenses with appropriate working distance.
  Answer: **a.** Performing retinoscopy on a child without cycloplegia.
**3. Clinical ophthalmology**
  The clinical finding of multiple, yellowish, ill-defined sub-retinal pigment epithelium (RPE) deposits, as seen in this image (Figure 1) from a patient with primary intraocular lymphoma (PIOL), most closely mimics the fundus appearance of which of the following acquired conditions, often leading to misdiagnosis?
  **Figure 1:**
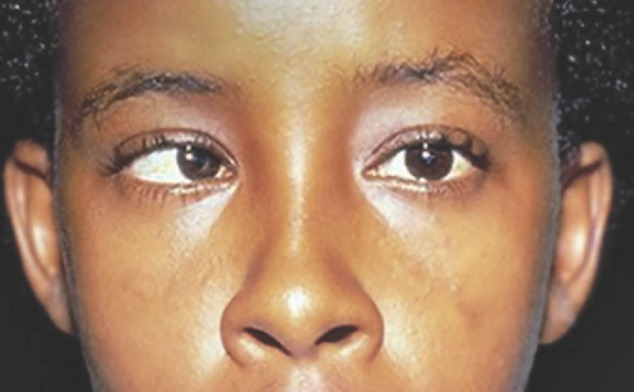
© OPHTHALMOLOGY FOUNDATION CC BY-NC-SA 4.0
  - Best’s vitelliform macular dystrophy
  - Acute retinal necrosis syndrome
  - Birdshot choroiditis
  - Retinal vein occlusion (RVO) sequelae
  Answer: **b.** Acute retinal necrosis syndrome.

The Ophthalmology Foundation team is pleased to support educators in developing high-quality assessment content and welcomes question submissions for review and feedback at exams@ophthalmologyfoundation.org.

**Figure:**
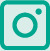
@ophthalmology.foundation

**Figure:**
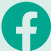
@Ophthalmology Foundation

**Figure:**
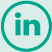
@Ophthalmology Foundation

**Figure:**
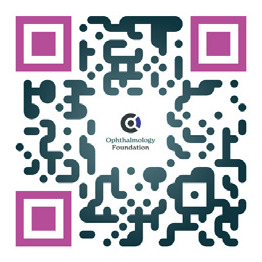


*The content of this page is supported by the Ophthalmology Foundation*

